# Mechanism of microplastics in respiratory disease from 2020 to 2024: visualization and bibliometric analysis

**DOI:** 10.3389/fmed.2025.1586772

**Published:** 2025-05-22

**Authors:** Zixi Du, Xia Yu, Xue Li, Ling Zhang, Yonghong Lin, Yulei He, Ying Wu

**Affiliations:** ^1^Department of Paediatrics, Chengdu Women’s and Children’s Central Hospital, School of Medicine, University of Electronic Science and Technology of China, Chengdu, China; ^2^Department of Clinical Laboratory, Chengdu Women’s and Children’s Central Hospital, School of Medicine, University of Electronic Science and Technology of China, Chengdu, China; ^3^Department of Obstetrics and Gynecology, Chengdu Women’s and Children’s Central Hospital, School of Medicine, University of Electronic Science and Technology of China, Chengdu, China

**Keywords:** microplastics, respiratory disease, mechanism, CiteSpace, bibliometric analysis

## Abstract

**Objective:**

The effect of microplastics (MPs) on the respiratory disease is extremely significant. Targeted intervention can be aided by recognizing the pathophysiology of microplastics in respiratory diseases. This research attempts to identify major trends in literatures and highlight key research based on bibliometric analysis to figure out present research areas and potential future research directions.

**Methods:**

Relevant academic works from 2020 to 2024 were downloaded from the Web of Science Core Collection (WoSCC). Subsequent examination of these records was performed utilizing multiple analytical tools: The R statistical package (version 4.3.2), CiteSpace software, the online analysis platform of Literature Metrology and the online interface of bibliometrix.^1^

**Results:**

A total of 78 qualified records were identified and included in the analysis. China has the highest number of publications. The most referenced journal was Science of the Total Environment. Chinese Academy of Sciences was the institution with the highest publication number. Toxicity, ingestion, accumulation, metabolism, gene, oxidative stress, inflammation and cell death were among the 25 most relevant terms.

**Conclusion:**

Research on the processes of MPs in the respiratory disease has advanced rapidly during the past 5 years. Human exposure (toxicity, ingestion, accumulation and metabolism), gene, oxidative stress, inflammation and cell death are the five main research area. In the following stage, deep studies on the connection of various mechanisms will be conducted, and efforts are expected to minimize the level of MPs in the human body, thus reducing the risk to humans.

## Introduction

The latest Global Burden of Disease Study suggested that in 2017, 544.9 million people globally had chronic respiratory diseases, a 39.8% increase from 1990 (1). With rapid economic growth and fast-paced life, humans are exposed to various sorts of microplastics (MPs), including plastic bottles, take-out food containers, clothes, etc. (2–4). MPs are pieces of plastic with a diameter of 5 mm or less, mainly including: polyethylene (PE), polypropylene (PP), polystyrene (PS) and polyvinyl chloride (PVC) (5–7). MPs have hazardous effects on respiratory system. Inhaling plastic particles increases the probability of getting respiratory disease, such as asthma, bronchitis, emphysema, fibrosis and so on (8–11). Notably, exposure risks exhibit geographical variations as the concentration of MPs in the air shows significant spatial differences. There is a gradual decrease in concentration levels transitioning from urban to rural to wildland sites (12). Moreover, populations in manufacturing zones may face increased health risks as a result of occupational exposure (13). Therefore, it is critical to elucidate the pathogenic mechanisms of MPs in the respiratory system for precise disease intervention.

Studies on the role of MPs in respiratory disease have been published by numerous authors worldwide. Thus, to assist researchers in studying the extensive literature related to this topic, promptly understand the general trend of this research, and carry out further studies in this domain, it is essential to collect information from relevant publications.

**Figure 1:**
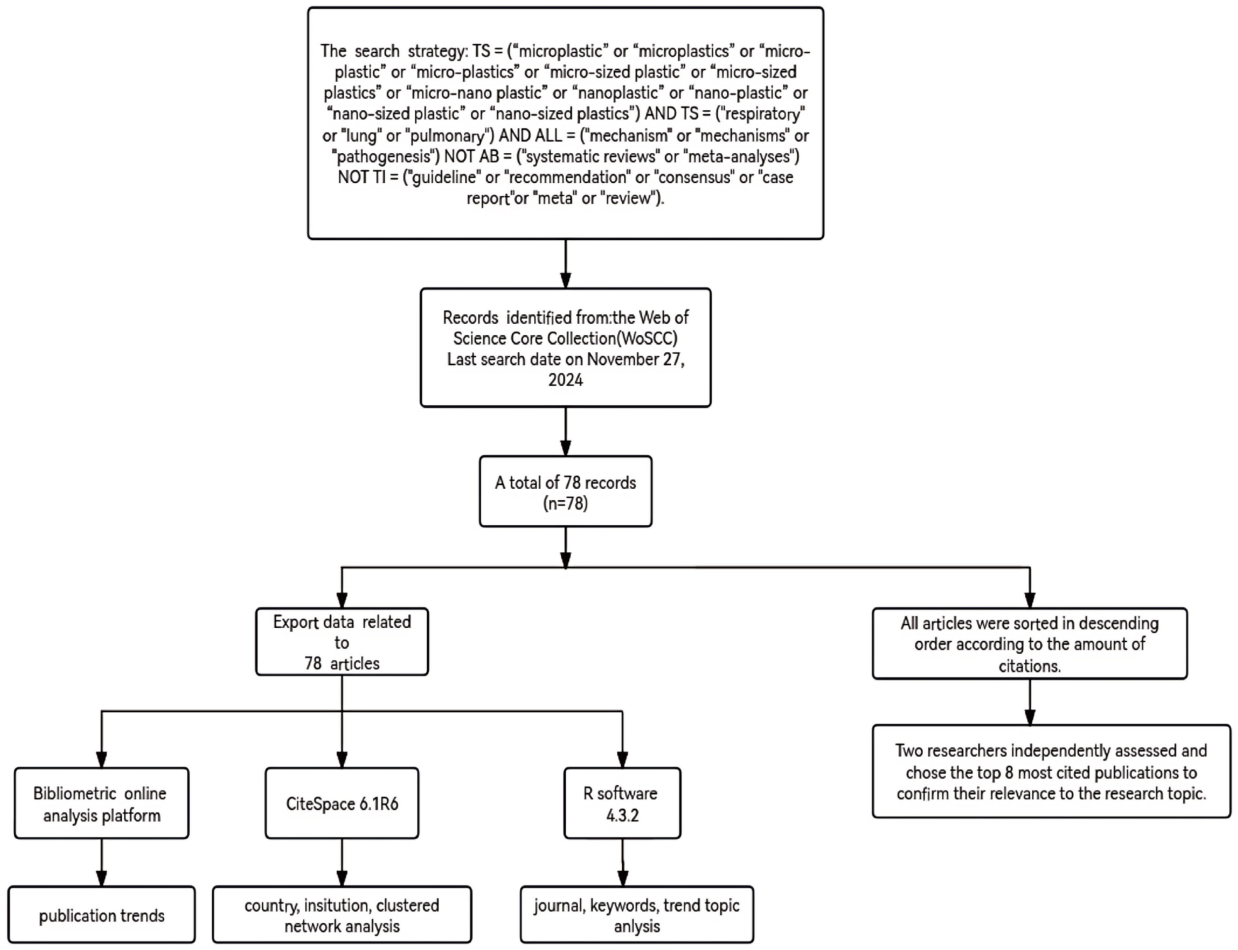
Database retrieval and data analysis process.

Bibliometric analysis is an approach of providing insight into the development tendency, research hotspots, and academic influence of a specific subject area through utilizing statistics and analyzing publication and citation data from scientific literature (14–16). And it has been frequently applied for reviewing vast amounts of various literature in the field of medicine (17–19). To undertake this study, as shown in Figure 1, CiteSpace (20, 21) was applied to recognize and visualize the data from the Web of Science Core Collection (WoSCC) from various perspectives, such as nations, institutions, co-cited authors, and co-cited references. Furthermore, trending topics and research hotspots were determined by using the “bibliometrix “R package and “biblioshiny” (22).

Currently, bibliometric analysis has not been applied to articles about the mechanisms of MPs in respiratory disease. In this work, we aimed to systematically review the latest research advancements in MPs related to the mechanisms of respiratory diseases, further synthesize the critical findings within this domain, and elucidate potential directions for future investigations.

## Materials and methods

### Data sources and search strategies

To ensure the academic quality and integrity of the research, we searched pertinent articles in the Social Sciences Citation Index (SSCI) and the Web of Science Core Collection (WoSCC) Science Citation Index Expanded (SCI-Expanded). The following search strategy was used: TS = (“microplastic” or “microplastics” or “micro-plastic” or “micro-plastics” or “micro-sized plastic” or “micro-sized plastics” or “micro-nano plastic” or “nanoplastic” or “nano-plastic” or “nano-sized plastic” or “nano-sized plastics”) AND TS = (“respiratory” or “lung” or “pulmonary”) AND ALL = (“mechanism” or “mechanisms” or “pathogenesis”) NOT AB = (“systematic reviews” or “meta-analyses”) NOT TI = (“guideline” or “recommendation” or “consensus” or “case report” or “meta” or “review”). Document Type was set to include “Articles” only from inception to the present (last search date on November 27, 2024).

### Data analysis and data visualization

All records indexed from WoSCC were saved to tab-delimited file and Bibtex format for “Full-text Records and References.” Following the initial data search, each article was evaluated independently by two researchers (ZD and XY) to guarantee that it was relevant to the study’s topic. Then, all the data were transferred into Bibliometrics’ Online Analysis Platform,^2^ where the “National total” option was applied to analyze the quantity of publications in each country and the “Total volume” option was applied to analyze the trends in publications over these years. Additionally, using the recorded TXT format data, the Bibliometrix package in R (Version 4.3.2) and a web interface for Bibliometrix called “biblioshiny” were used to determine the most often referenced documents as well as the most common word and trend topics. After importing the TXT format files into CiteSpace program, we set the following parameters: the time span (2020–2024), years per slice (1), links (strength: cosine, scope: within slices), selection criteria (g-index: *k* = 25, Top *N* = 50, Top *N*% = 10%, maximum number of selected items per slice = 100), and the default settings for each other parameter were maintained.

### Quality control of bibliometric analysis

In our research, we took the following quality control measures: 1. Data cleaning: We processed the data, removed redundant and erroneous data, and ensured that all data was in a consistent format to improve the accuracy of the analysis. 2. Tool calibration: We used multiple analysis tools and calibrated the output results by comparing them with existing standards and data sets. 3. Sample representativeness: When selecting literature data, we ensured that sufficient sample time periods were included to maintain the representativeness of the results. 4. Result verification: Our results have been cross-verified, multiple independent analysis to ensure reliability. In addition, we invited experts in the field to review our results to ensure their validity. 5. Transparency: We have documented the methodological process in detail so that future researchers can repeat our analysis.

## Results

### Analysis of annual growth trend

**Figure 2:**
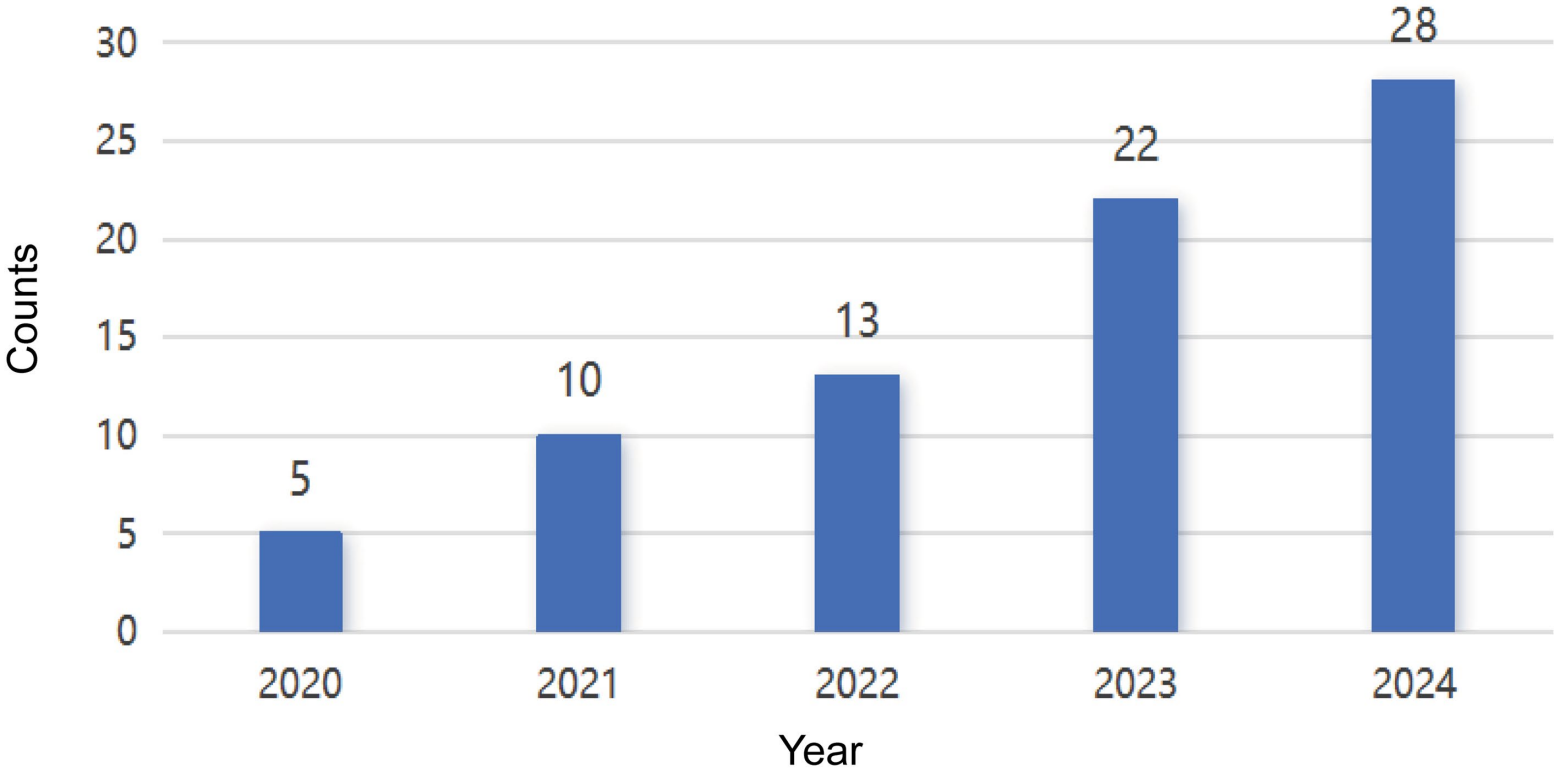
The number of annual publications on the research progress on the mechanism of microplastics in respiratory system and the growth trend from the beginning to November 2024.

In total, we incorporated 78 articles which were eligible for inclusion. Then we imported these data on the Bibliometrics Online Analysis Platform (see text footnote 2). These 78 articles were organized in descending order based on their citation counts when analyzing the Most Local Cited Documents. We observed that beginning with the ninth article “Microplastic consumption and physiological response in *Acartia clausi* and *Centropages typicus*: Possible roles of feeding mechanisms,” the citation count for each subsequent article was consistently one. Consequently, in the final discussion section, we limited in review our analysis to the top 8 most locally cited articles to synthesize the primary research contents and identify key trends in recent years (Figure 1). The number of articles published per year were illustrated on Figure 2, which continued to grow steadily. In 2020, there were only 5 published papers. However, the number of articles reached more than 20 since 2023. It is obvious to see that the impact of MPs on respiratory disease is receiving great attention.

### Country analysis

**Figure 3:**
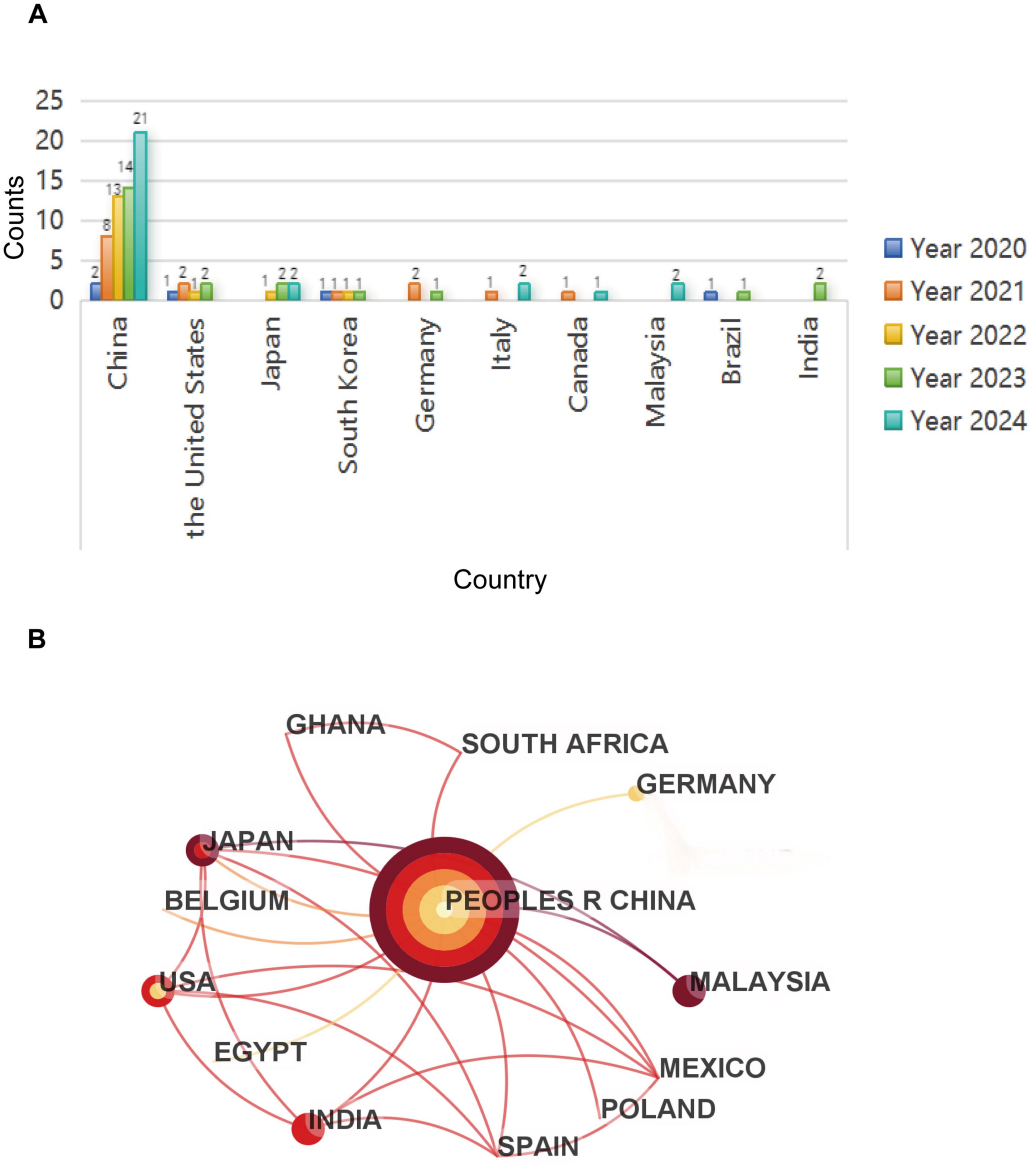
A collaboration to study the number of publications on the mechanism of microplastics in respiratory system in different countries **(A)** Number and growth trends in annual publications in the top 10 countries; **(B)** Inter-country cooperation. Each circle represents a country. The size of the circle is positively correlated with the number of articles published by the country, and the link between the two circles indicates the cooperation of the two countries on the same article.

Publications from different countries conducting research in this field from 2020 to 2024 were further analyzed. If an article involves contributions from authors across multiple countries, it may lead to duplicate counting. For instance, the number of publications in 2023 exceeded 22. In Figure 3A, the top 10 countries were showed on a bar chart. These articles were scattered in Brazil, Canada, India, Malaysia, Germany, Italy, South Korea, Japan, the United States and China. China ranked first in terms of the number of publications, which also had the most citation (*n* = 1938). In addition, it is worth noting that although Indonesia is not one of the top 10 countries, the citation of it was 179, which placed second to China. This suggests that it receives great recognition for its article as well. Then, a cooperation network based on the cross-country links was constructed through CiteSpace program. There were 52 connections between 29 countries that have formed partnerships. Among these, China was the country that had established the most cooperative relationships, with a total of 12 countries involved in these partnerships. Thus, Figure 3B specifically illustrated the collaborative network of China alone.

### Journal analysis

All of these 78 articles appeared in 38 distinct journals from 2020 to 2024. The bibliometric online analysis was used to assess the impact of the journals. The 10 most referenced journals were enumerated on Table 1. Five of the publishers were located in United Kingdom, three in the United States and two in Netherlands. Journal of Hazardous Materials was recognized as a pioneer in the domain with impact factor (IF) of 12.2. And Science of the Total Environment had the highest citations (424) with an IF of 8.2.

**Table 1:** The top 10 most referenced journals that published articles on mechanism of microplastics in respiratory disease (sorted by total citation).

Rank	Journal title	Country	Total citations	Impact factor (2023)
1	Science of the Total Environment	Netherlands	424	8.2
2	Journal of Hazardous Materials	Netherlands	291	12.2
3	Environmental Science & Technology	United States	282	10.9
4	Environmental Pollution	United Kingdom	279	7.6
5	Marine Pollution Bulletin	United Kingdom	145	5.3
6	Chemosphere	United Kingdom	141	8.1
7	Environment International	United Kingdom	117	10.3
8	Ecotoxicology and Environmental Safety	United States	81	6.2
9	Scientific Reports	United Kingdom	67	3.8
10	Environmental Research	United States	62	7.7

### Institution analysis

**Figure 4:**
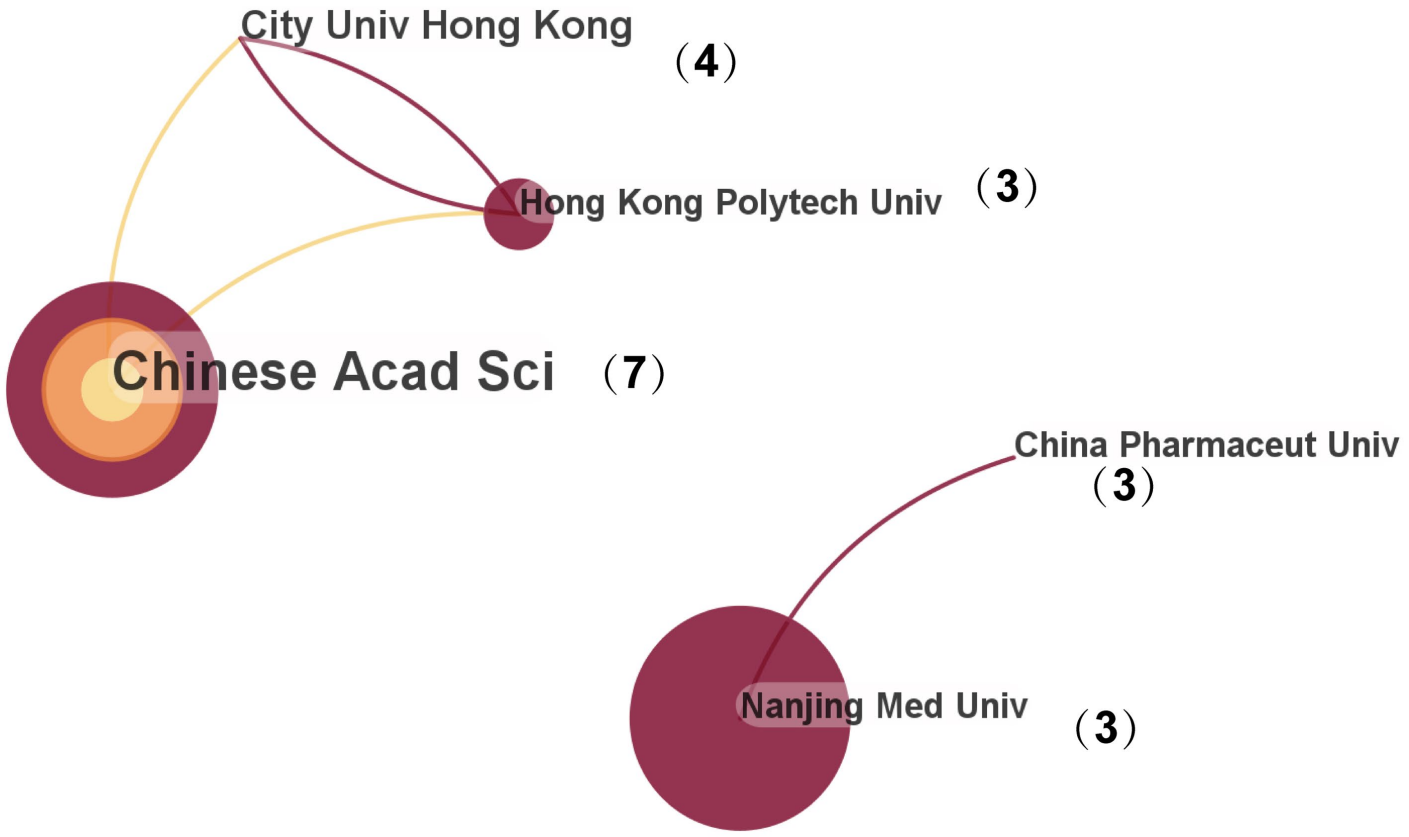
Cooperation between institutions. Each circle represents an institution. The size of each circle is not only correlated with the number of publications by the institution, but also with the extent of collaborative relationships between the institution and others.

We analyzed the number of publications and how effectively they cooperated through Citespace. Totally, 110 nodes and 130 linkages were identified after assessment of collaborations between different institutions. And the top 5 most productive institutions were shown on Figure 4, which were located in China. Chinese Academy of Sciences was the most productive (article number = 7) and it collaborates with the most organizations (15). City University of Hong Kong was the second most productive institution. China Pharmaceutical University, Nanjing Medical University and The Hong Kong Polytechnic University had three articles respectively. Notably, The Hong Kong Polytechnic University has established relationships with 13 institutions, second only to Chinese Academy of Sciences. We look forward to more transnational collaborations between institutions in the future, prompting further research on mechanism of MPs in disease.

### Clustered network in co-analysis

**Figure 5:**
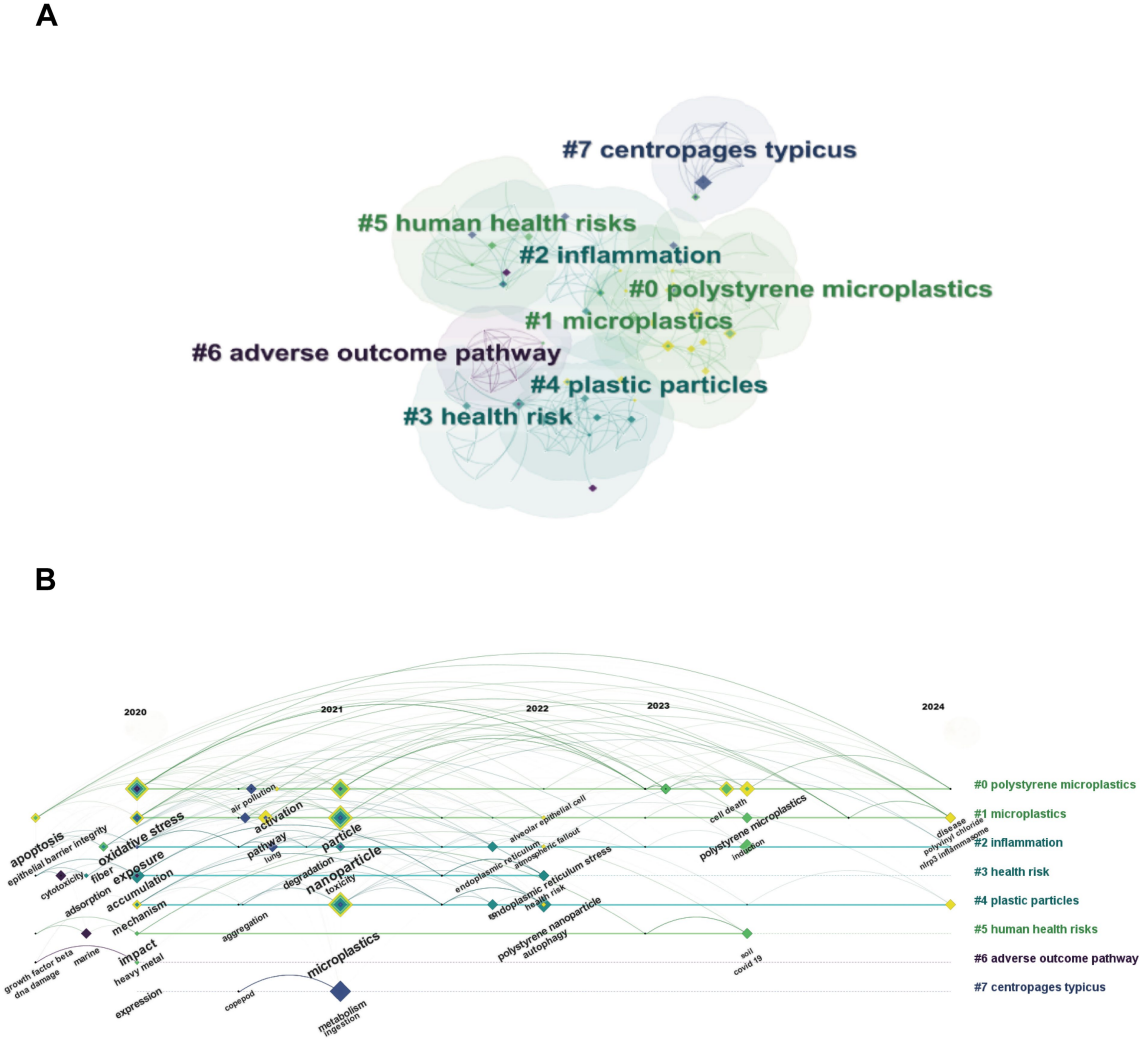
Cluster network analysis of reference co-citation **(A)** A cluster network of co-citation status for references and cited articles via CiteSpace. Displays the top 8 largest clusters of the referenced article; **(B)** Timeline view of the top 8 clusters of the article is cited.

A cluster network analysis was performed to study the co-cited papers in more detail. The 78 articles were classified into several groups in accordance with the logical outcome of the homogeneity analysis, which indicates that two publications are more likely to be alike if they possess a considerable number of references. After the “Show the Largest *K* Clusters” node (*K* = 25) was selected, 10 noteworthy clusters were identified from 150 keywords. Including #0 polystyrene microplastics, #1 microplastics, #2 inflammation, #3 health risk, #4 plastic particles, #5 human health risks, #6 adverse outcome pathway, #7 *centropages typicus* (Figure 5A). In addition, we drew a timeline to display highly-discussed studies and the citation tree rings of various sizes (Figure 5B).

Oxidative stress and human exposure have been key concerns in the research regarding the mechanism of MPs in respiratory disease since 2020. And from 2021, the role of different size of MPs particles have been gradually gaining attention. Furthermore, since 2022, researchers have increasingly directed their attention toward cellular-level studies, including endoplasmic reticulum stress, autophagy and so on. By 2023, cell death has become a research hotpot. In addition, NIPR3 inflammasome was proposed firstly in 2024. And polyvinyl chloride, a type of MPs predominantly utilized in electrical insulation materials, started to garner attention.

### Keywords analysis

**Figure 6:**
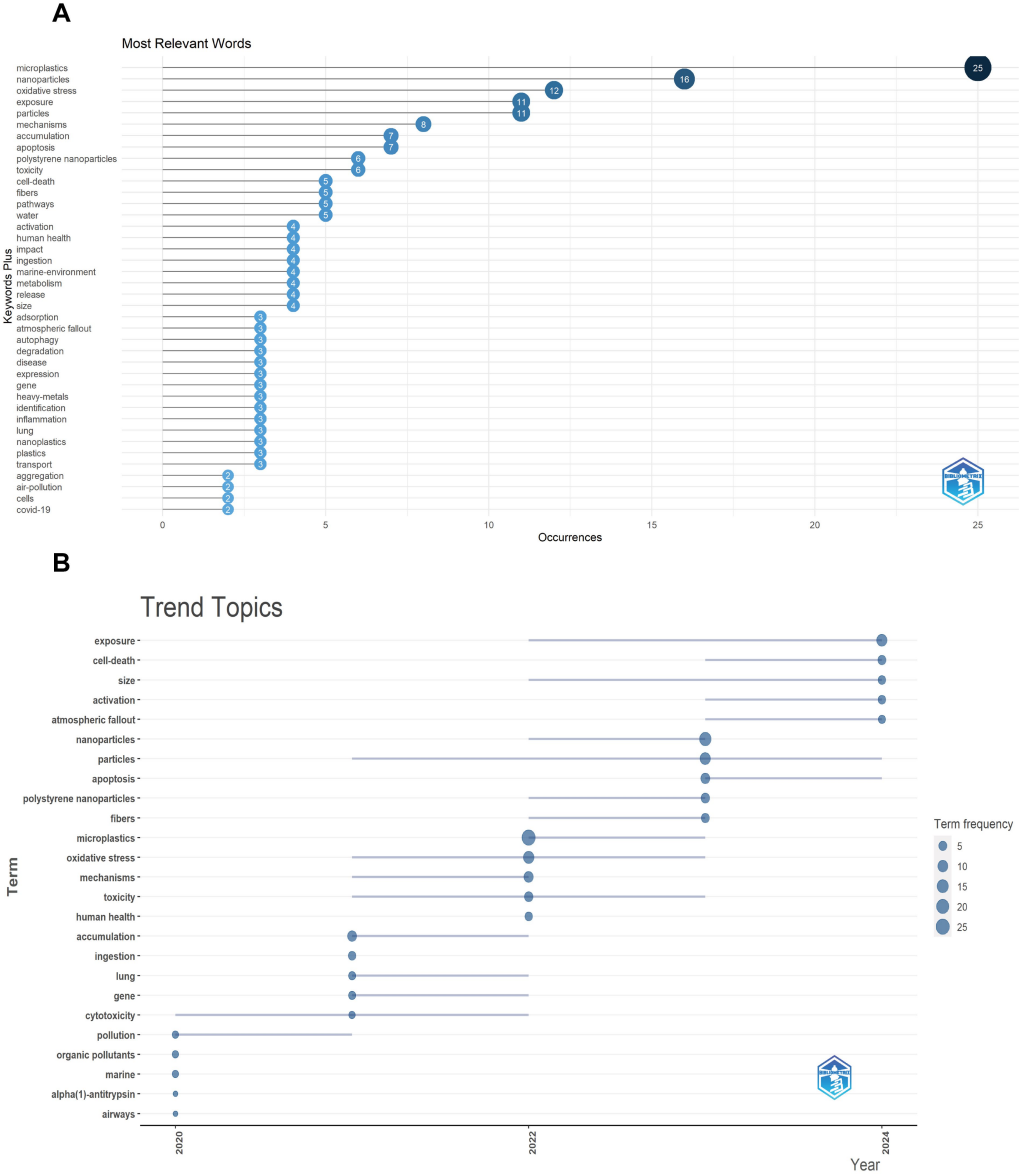
Analysis of keywords **(A)** The most relevant words in the top 40; **(B)** Trend topics are shown.

Through analyzing the keywords, we can uncover the research hotspots. After removing search-related terms and general words, such as “microplastics,” “nanoparticles,” “exposure,” “particles,” “mechanisms,” “polystyrene nanoparticles,” the top 10 keywords were oxidative stress, accumulation, apoptosis, toxicity, cell death, ingestion, metabolism, autophagy, gene and inflammation (Figure 6A). Then, we analyzed the trend topics on Bibliometric platform as well to learn about the research focus. Cell death got attention again in the last 2 years and it had become a hotspot rapidly (Figure 6B).

## Discussion

In total, we retrieved 78 research articles on the mechanisms of MPs in respiratory disease from 2020 to 2024 and presented a visual citation analysis. And we used three analytical tools to obtain a comprehensive picture was from different perspectives. Since 2023, the number of relevant publications began to increase rapidly, which suggested that the respiratory hazards of MPs had gained great attention. In addition, a systematic picture of the field during the last 5 years has been presented by conducting journal, keyword, intercountry, and interinstitutional studies.

The contents of the 8 most locally cited pieces, which have been significant in the field’s evolution, were examined in order to provide a more profound comprehension of the most frequently cited studies (Table 2). By synthesizing clustered network and keywords, four points were summed up: human exposure (toxicity, ingestion, accumulation and metabolism), gene, oxidative stress and inflammation, cell death.

**Table 2:** The top 8 most locally cited papers on mechanism of microplastics in respiratory disease during 2020 to 2024.

Rank	Title	First Author	Local Citation	Global Citation	DOI	Year
1	Polystyrene microplastic particles: In vitro pulmonary toxicity assessment	Dong CD	14	347	10.1016/j.jhazmat.2019.121575	2020
2	Toxic effects of nanoplastics with different sizes and surface charges on epithelial-to-mesenchymal transition in A549 cells and the potential toxicological mechanism	Halimu G	10	85	10.1016/j.jhazmat.2022.128485	2022
3	In vitro evaluation of nanoplastics using human lung epithelial cells, microarray analysis and co-culture model	Yang S	7	88	10.1016/j.ecoenv.2021.112837	2021
4	Detrimental effects of microplastic exposure on normal and asthmatic pulmonary physiology	Lu K	6	85	10.1016/j.jhazmat.2021.126069	2021
5	Polypropylene nanoplastic exposure leads to lung inflammation through p38-mediated NF-κB pathway due to mitochondrial damage	Woo JH	3	51	10.1186/s12989-022-00512-8	2023
6	Bioaccumulation of differently-sized polystyrene nanoplastics by human lung and intestine cells	Zhang YX	2	39	10.1016/j.jhazmat.2022.129585	2022
7	Polystyrene microplastic particles induce autophagic cell death in BEAS-2B human bronchial epithelial cells	Jeon MS	2	28	10.1002/tox.23705	2023
8	Inhalation exposure to polystyrene nanoplastics induces chronic obstructive pulmonary disease-like lung injury in mice through multi-dimensional assessment	Yang S	2	5	10.1016/j.envpol. 2024.123633	2024

### Human exposure (toxicity, ingestion, accumulation and metabolism)

The earliest article among the 8 articles on pulmonary toxicity was published in 2000. Dong et al. discovered that in BEAS-2B cells, polystyrene microplastics (PS-MPs) can induce reactive oxygen species (ROS) generation, which may result in cytotoxic and inflammatory effects (23). In addition, the discovery of distributions of MPs particles and macrophages co-labeled within and outside of bronchioles by Lu et al. proved ingestion of MPs particles by macrophages (24). Then, in 2022, Zhang et al. studied the effect of the physicochemical properties of MPs on bioaccumulation (25). Surface area was demonstrated to play an important role in the interaction of polystyrene nanoplastics (PS-NPs) with cell membranes. Moreover, the accumulation of positively charged PS-MPs (NH2-PS-MP) in lysosomes and the deformation of the nucleus were noticed in BEAS-2B cells when autophagy was stimulated (26). Studies have demonstrated that the application of probiotics and other microorganisms for the degradation of MPs can significantly mitigate the accumulation of MPs within organisms (27). Furthermore, in an experiment conducted by Halimu et al., it was discovered that PS-NPs could disrupt the function of mitochondria, which was indicated by changes in membrane potential and the reduction of cellular energy metabolism (28).

### Gene

During the examination of 8 articles, it was found that 3 of them centered on gene. According to a study published in 2021, Yang et al. discovered that in BEAS-2B cells, exposure to PS-NPs modified the gene expression in concentration-dependent form through microarray detection. 770 genes in the 7.5 μg/cm^2^ group and 1951 genes in the 30 μg/cm^2^ group were significantly altered compared with the control group (29). Over the same period, Lu et al. pointed out that MPs exposure affected gene clusters connected with immune reactivity, cellular stress reaction, and regulated cell death (24). In a subsequent study, Halimu et al. found that following PS-NP treatment, the interference with the NADPH oxidase 4 gene notably alleviated the impairment to oxidative phosphorylation and transmembrane potential in mitochondria, thereby being capable of inhibiting epithelial-to-mesenchymal transition in A549 cells (28).

### Oxidative stress and inflammation

Among these 8 articles, 6 indicated that oxidative stress and inflammation were closely associated with respiratory disease caused by MPs. The earliest article was published in 2020. High levels of PS-MPs may increase the expression of IL-8 and IL-6, which lead to oxidative stress and stimulate the immune system. Dong et al. observed that the accumulation of ROS significantly rose merely under the PS-MPs concentration of at 1000 μg/cm^2^. The formation of massive ROS exceeded the capacity of cellular antioxidant enzymes, resulting in significant oxidative stress. Mirroring the ROS trajectory, intensified HO-1 (a ubiquitous stress-response protein) expression was quantified in BEAS-2B cells under high-concentration PS-MP treatment (23). Also, Yang et al. found that after 24-h exposure, PS-NPs enhanced the production of ROS and the content of superoxide anion in pulmonary epithelial cells. Moreover, following PS-NPs treatment, pulmonary cells demonstrated a dose-responsive decline in the enzymatic activities of GSH-Px, CAT and SOD, which were also indices of oxidative stress. Apart from the expression of IL-6, they further observed rise of MCP-1 (a kind of proinflammatory mediator) in lung cells. However, PS-NPs did not significantly affect TNF-*α* levels in lung cells, regardless of concentration. And ICAM-1, a type of cell adhesion molecule, was enhanced significantly (29). On the base of that, they proved that the exposure to PS-NPs altered the components of inflammatory cells in bronchoalveolar lavage fluid. Macrophages continued to be the predominant cell type in BALF following PS-NPs exposure regardless of the duration, yet the percentage of macrophages decreased, accompanied by a accordant rise in neutrophils and leukomonocyte (30).

Furthermore, in 2023, Woo et al. pointed out the ROS production caused by PP stimulation contributed to the activation of NF-κB through p38 phosphorylation. The production of ROS and levels of antioxidase such as catalase, superoxide dismutase (SOD)1, SOD2, and glutathione peroxidase-1 were markedly elevated in A549 cells exposed to PP nanoplastic. However, the protein levels of the endoplasmic reticulum (ER) stress indicators remained unchanged. Additionally, PP-exposed A549 cells had considerably higher amounts of NRF2, which may influenced the level of antioxidant proteins that prevent oxidative damage (31). Subsequently, in a study conducted by Jeon et al., differently charged PS-MPs were used. NH2-PS-MPs induced higher ROS generation. And the alterations in ER stress markers were also measured. Phosphorylated PERK and phosphorylated IRE1 increased dose-dependently after exposed to NH2-PS-MPs. Meanwhile, the expression of p-eIF2α and ATF4 were upregulated (26). More than that, Lu et al. noted that, through nasal MPs exposure, the levels of eosinophil and lymphocyte in asthmatic mice were higher than those in normal mice treated with saline. Meanwhile, higher levels of both IgE and IgG1 were detected in this group (24).

### Cell death

In the study conducted by Yang et al., the levels of cleaved-caspase9 and cleaved-caspase3 were markedly increased in cells treated with PS-NPs, which were apoptosis-related markers. Moreover, the proportion of BCL2/BAX was notably reduced, indicating that the anti-apoptotic ability was weakened. In a dose-dependent way, the amount of LDH produced from the cells treated with PS-NPs increased greatly. Meanwhile, a dramatic reduction in the level of proliferating cell nuclear antigen and cyclin D1 was observed, which were established marker of cell proliferation (29). On this basis, they further found ferroptosis might be the latent mechanism underlying the lung injury caused by PS-NPs in 2024. Ferroptosis represents a type of cellular death, and it is often accompanied by mitochondrial impairment, lipid peroxidation, and iron accumulation (32). The ferroptosis pathway was activated by PS-NPs in both the high-dose subacute and subchronic exposure groups. And the expression of GPX4, NCOA4, and Ferritin in lung tissue declined following exposure to various doses of PS-NPs (30).

Then, in 2021, Lu et al. pointed out that the existence of MPs modified protein homeostasis and stress response, which might result in cell apoptosis. MPs led to the enhanced expression of transmembrane receptors. Additionally, the genes related to immune cells, such as IgD and BTLA, were equally up-regulated. This was related to the suppression of a group of heat shock proteins, such as HSPA8, HSP90AA1, DNAJB1 and so on, as well as the suppression of STIP1. The majority of these gene dysregulations are related to programmed cell death, lymphocyte generation and leucopoiesis (24). The research carried out by Jeon et al. demonstrated that NH2-PS-MPs triggered autophagy in BEAS-2B cells through the elevation of p62 and LC-3 expression. The indication of ER stress proteins demonstrated that NH2-PS-MPs aggravated endoplasmic reticulum stress via the PERK-EIF2α and ATF4-CHOP pathways (26).

Although there has been great progress in the research on the mechanism of MPs in respiratory system, the following issues need to be considered: First, it is vital to study the synergistic or antagonistic effects of MPs with other air pollutants, microbes, and allergens on the development of respiratory disease. Second, whether exposure to MPs leads to abnormal expression of genes related with respiratory disease by affecting epigenetic pathways needs further inquiry. Third, it is essential to expand the research subjects, including children, pregnant women, the elderly, people with compromised immunity, and people with occupational exposure. Fourth, multi-omics techniques need to be applied to discover new biomarkers and potential therapeutic targets (33). Fifth, effective interventions, such as biologics, immunomodulation and other approaches ought to be explored to alleviate lung injury caused by MPs.

The constraints of the present study need to be taken into consideration. The information was gathered from a single database. Issues like delayed data updates could lead to deviations in final results. With the aim of achieving a more profound comprehension of this research topic, multiple methods will be used in subsequent studies.

## Conclusion

Research on the processes of MPs in respiratory disease has advanced rapidly during the past 5 years. Human exposure (toxicity, ingestion, accumulation and metabolism), gene, oxidative stress, inflammation and cell death are the five main research area. MPs could induce pulmonary toxicity via multifaceted mechanisms. Key pathways involve ROS-mediated oxidative stress, inflammatory cytokine activation (e.g., IL-6, IL-8, MCP-1), and mitochondrial dysfunction characterized by altered membrane potential and impaired energy metabolism. The physicochemical properties of MPs (e.g., surface charge, size) significantly affect cellular interactions, lysosomal accumulation, and damage to nucleus or organelles. Genomic analyses indicate that MPs disrupt genes associated with immune responses, oxidative stress, and cell death, while also triggering apoptosis, ferroptosis and autophagy-related ER stress. Additionally, MPs alter immune cell profiles (e.g., increased neutrophils, decreased macrophages) and elevate levels of allergic mediators. And interventions such as probiotics or modulation of antioxidant pathways exhibit potential in reducing MP-induced toxicity. In the future, various analytical methods should be used to investigate the relevant mechanisms from different perspectives and propose efficient strategies to reduce the injury caused by MPs to the human body.

## Data Availability

The data supporting the findings of this research are available in the Web of Science database, as mentioned in the manuscript’s Methods section.

## Footnotes

^2^ http://bibliometric.com

## References

[1] GBD Chronic Respiratory Disease Collaborators. Prevalence and attributable health burden of chronic respiratory diseases, 1990-2017: a systematic analysis for the global burden of disease study 2017. Lancet Respir Med. (2020) 8:585–96. doi: 10.1016/S2213-2600(20)30105-3, PMID: 32526187 PMC7284317

[2] Shruti VC Pérez-Guevara F Elizalde-Martínez I Kutralam-Muniasamy G. First study of its kind on the microplastic contamination of soft drinks, cold tea and energy drinks - future research and environmental considerations. Sci Total Environ. (2020) 726:138580. doi: 10.1016/j.scitotenv.2020.138580, PMID: 32315857

[3] Vianello A Jensen RL Liu L Vollertsen J. Simulating human exposure to indoor airborne microplastics using a breathing thermal manikin. Sci Rep. (2019) 9:8670. doi: 10.1038/s41598-019-45054-w, PMID: 31209244 PMC6573036

[4] Jamil A Ahmad A Irfan M Hou X Wang Y Chen Z . Global microplastics pollution: a bibliometric analysis and review on research trends and hotspots in agroecosystems. Environ Geochem Health. (2024) 46:486. doi: 10.1007/s10653-024-02274-y 39509054

[5] Alimba CG Faggio C. Microplastics in the marine environment: current trends in environmental pollution and mechanisms of toxicological profile. Environ Toxicol Pharmacol. (2019) 68:61–74. doi: 10.1016/j.etap.2019.03.001, PMID: 30877952

[6] Gan Q Cui J Jin B. Environmental microplastics: classification, sources, fates, and effects on plants. Chemosphere. (2023) 313:137559. doi: 10.1016/j.chemosphere.2022.137559, PMID: 36528162

[7] Wang C Zhao J Xing B. Environmental source, fate, and toxicity of microplastics. J Hazard Mater. (2021) 407:124357. doi: 10.1016/j.jhazmat.2020.124357 33158648

[8] Chen C Liu F Quan S Chen L Shen A Jiao A . Microplastics in the Bronchoalveolar lavage fluid of Chinese children: associations with age, City development, and disease features. Environ Sci Technol. (2023) 57:12594–601. doi: 10.1021/acs.est.3c01771, PMID: 37578997

[9] Huang S Huang X Bi R Guo Q Yu X Zeng Q . Detection and analysis of microplastics in human sputum. Environ Sci Technol. (2022) 56:2476–86. doi: 10.1021/acs.est.1c03859, PMID: 35073488

[10] Li X Zhang T Lv W Wang H Chen H Xu Q . Intratracheal administration of polystyrene microplastics induces pulmonary fibrosis by activating oxidative stress and Wnt/β-catenin signaling pathway in mice. Ecotoxicol Environ Saf. (2022) 232:113238. doi: 10.1016/j.ecoenv.2022.113238, PMID: 35121255

[11] Sartorelli P d'Hauw G Spina D Volterrani L Mazzei MA. A case of hypersensitivity pneumonitis in a worker exposed to terephthalic acid in the production of polyethylene terephthalate. Int J Occup Med Environ Health. (2020) 33:119–23. doi: 10.13075/ijomeh.1896.01465, PMID: 31691678

[12] Luo D Wang Z Liao Z Chen G Ji X Sang Y . Airborne microplastics in urban, rural and wildland environments on the Tibetan plateau. J Hazard Mater. (2024) 465:133177. doi: 10.1016/j.jhazmat.2023.133177, PMID: 38064947

[13] Tanjil RH Islam MS Islam Z Roy S Nahian S Salam A. Atmospheric microplastic pollution in textile industrial areas: source, composition, and health risk assessment. Bull Environ Contam Toxicol. (2025) 114:51. doi: 10.1007/s00128-025-04021-0, PMID: 40119914

[14] Manoj Kumar L George RJ SA P. Bibliometric analysis for medical research. Indian J Psychol Med. (2023) 45:277–82. doi: 10.1177/02537176221103617, PMID: 37152388 PMC10159556

[15] Wang S Zhou H Zheng L Zhu W Zhu L Feng D . Global trends in research of macrophages associated with acute lung injury over past 10 years: a bibliometric analysis. Front Immunol. (2021) 12:669539. doi: 10.3389/fimmu.2021.669539, PMID: 34093568 PMC8173163

[16] Agarwal A Durairajanayagam D Tatagari S Esteves SC Harlev A Henkel R . Bibliometrics: tracking research impact by selecting the appropriate metrics. Asian J Androl. (2016) 18:296–309. doi: 10.4103/1008-682x.171582, PMID: 26806079 PMC4770502

[17] Garbern SC Hyuha G González Marqués C Baig N Chan JL Dutta S . Authorship representation in global emergency medicine: a bibliometric analysis from 2016 to 2020. BMJ Glob Health. (2022) 7:9538. doi: 10.1136/bmjgh-2022-009538, PMID: 35760436 PMC9237874

[18] Jiang S Liu Y Zheng H Zhang L Zhao H Sang X . Evolutionary patterns and research frontiers in neoadjuvant immunotherapy: a bibliometric analysis. Int J Surg. (2023) 109:2774–83. doi: 10.1097/js9.0000000000000492, PMID: 37216225 PMC10498839

[19] Liu M Liu J Xiong F Xu K Pu Y Huang J . Research advances of microplastics and potential health risks of microplastics on terrestrial higher mammals: a bibliometric analysis and literature review. Environ Geochem Health. (2023) 45:2803–38. doi: 10.1007/s10653-022-01458-8, PMID: 36598611 PMC9811881

[20] Chen C Song M. Visualizing a field of research: a methodology of systematic scientometric reviews. PLoS One. (2019) 14:e0223994. doi: 10.1371/journal.pone.0223994, PMID: 31671124 PMC6822756

[21] Wei N Xu Y Li Y Shi J Zhang X You Y . A bibliometric analysis of T cell and atherosclerosis. Front Immunol. (2022) 13:948314. doi: 10.3389/fimmu.2022.948314, PMID: 36311729 PMC9606647

[22] Cao Y Dong Q Wang D Zhang P Liu Y Niu C. MicrobiomeMarker: an R/Bioconductor package for microbiome marker identification and visualization. Bioinformatics. (2022) 38:4027–9. doi: 10.1093/bioinformatics/btac438, PMID: 35771644

[23] Dong CD Chen CW Chen YC Chen HH Lee JS Lin CH. Polystyrene microplastic particles: in vitro pulmonary toxicity assessment. J Hazard Mater. (2020) 385:121575. doi: 10.1016/j.jhazmat.2019.121575, PMID: 31727530

[24] Lu K Lai KP Stoeger T Ji S Lin Z Lin X . Detrimental effects of microplastic exposure on normal and asthmatic pulmonary physiology. J Hazard Mater. (2021) 416:126069. doi: 10.1016/j.jhazmat.2021.126069, PMID: 34492895

[25] Zhang YX Wang M Yang L Pan K Miao AJ. Bioaccumulation of differently-sized polystyrene nanoplastics by human lung and intestine cells. J Hazard Mater. (2022) 439:129585. doi: 10.1016/j.jhazmat.2022.129585, PMID: 35850063

[26] Jeon MS Kim JW Han YB Jeong MH Kim HR Sik Kim H . Polystyrene microplastic particles induce autophagic cell death in BEAS-2B human bronchial epithelial cells. Environ Toxicol. (2023) 38:359–67. doi: 10.1002/tox.23705, PMID: 36485005

[27] Bozkurt HS Yörüklü HC Bozkurt K Denktas C Bozdogan A Özdemir O . Biodegradation of microplastic by probiotic bifidobacterium. Int J Global Warm. (2022) 26:429–43. doi: 10.1504/IJGW.2022.122435

[28] Halimu G Zhang Q Liu L Zhang Z Wang X Gu W . Toxic effects of nanoplastics with different sizes and surface charges on epithelial-to-mesenchymal transition in A549 cells and the potential toxicological mechanism. J Hazard Mater. (2022) 430:128485. doi: 10.1016/j.jhazmat.2022.128485, PMID: 35739668

[29] Yang S Cheng Y Chen Z Liu T Yin L Pu Y . In vitro evaluation of nanoplastics using human lung epithelial cells, microarray analysis and co-culture model. Ecotoxicol Environ Saf. (2021) 226:112837. doi: 10.1016/j.ecoenv.2021.112837, PMID: 34619472

[30] Yang S Zhang T Ge Y Yin L Pu Y Liang G. Inhalation exposure to polystyrene nanoplastics induces chronic obstructive pulmonary disease-like lung injury in mice through multi-dimensional assessment. Environ Pollut. (2024) 347:123633. doi: 10.1016/j.envpol.2024.123633 38423272

[31] Woo JH Seo HJ Lee JY Lee I Jeon K Kim B . Polypropylene nanoplastic exposure leads to lung inflammation through p38-mediated NF-κB pathway due to mitochondrial damage. Part Fibre Toxicol. (2023) 20:2. doi: 10.1186/s12989-022-00512-8, PMID: 36624477 PMC9829531

[32] Li J Cao F Yin HL Huang ZJ Lin ZT Mao N . Ferroptosis: past, present and future. Cell Death Dis. (2020) 11:88. doi: 10.1038/s41419-020-2298-2, PMID: 32015325 PMC6997353

[33] Liang D Wang Y Qian K. Nanozymes: applications in clinical biomarker detection. Interdiscip Med. (2023) 1:e20230020. doi: 10.1002/inmd.20230020

